# Interleukin-1β Gene (*IL-1B*) rs16944 (-511 C > T) Promoter Polymorphism Is Associated with Cutaneous Melanoma Susceptibility, Stage, and Anatomical Localization in a Northern Italian Case–Control Study

**DOI:** 10.3390/curroncol33070436

**Published:** 2026-07-22

**Authors:** Sabina Cauci, Cinzia Buligan, Patrizia Nacci, Gianluca Petris, Giuseppe Stinco

**Affiliations:** 1 Department of Medicine, University of Udine, Piazzale Kolbe 4, 33100 Udine, Italygiuseppe.stinco@uniud.it (G.S.); 2 Institute of Dermatology, Azienda Sanitaria Universitaria Friuli Centrale (ASUFC), Santa Maria della Misericordia, Friuli Centrale University Healthcare Hospital of Udine, 33100 Udine, Italy; 3 Fondazione Italiana Fegato ETS—Italian Liver Foundation ETS, 34149 Trieste, Italy

**Keywords:** malignant melanoma, skin cancer, metastasis, cytokine IL-1 beta, innate immunity, inflammation, *IL-1B* gene SNP, promoter, genetic variation, anti-tumor activities, cancer susceptibility

## Abstract

Immunity plays critical roles in cutaneous melanoma. We investigated the association between the pro-inflammatory interleukin-1β gene (*IL-1B*) rs16944 (-511 C > T) promoter single nucleotide polymorphism (SNP) and cutaneous melanoma susceptibility and clinical features. This observational case–control study included 133 patients with cutaneous melanoma and 900 healthy controls from Northeastern Italy. The rs16944 C > T polymorphism was determined by genomic DNA restriction fragment analysis. The *IL-1B* rs16944 C allele (OR = 1.44, *p* = 0.014) and CC genotype (OR = 1.48, *p* = 0.037) were associated with modestly higher odds of melanoma. Among melanoma patients, the CC genotype was associated with Stage I disease (OR = 2.45, *p* = 0.016) and Breslow thickness ≤ 0.75 mm (OR = 2.27, *p* = 0.049), whereas it was less frequent in Stage IV melanoma (OR = 0.37, *p* = 0.029). The CT genotype was associated with Stage IV melanoma (OR = 3.03, *p* = 0.014) and with lower-limb (OR = 2.45, *p* = 0.038) and lower-extremity (OR = 3.09, *p* = 0.005) melanoma. These divergent associations are exploratory and do not establish disease trajectory or a functional effect of rs16944; mechanistic interpretation remains uncertain because IL-1β expression or activity was not measured in this cohort. To our knowledge, this is the first report of an association between a genetic polymorphism and lower-limb/lower-extremity melanoma. These exploratory findings require confirmation in independent cohorts.

## Simple Summary

We determined the genotype and allele frequencies of the interleukin-1β gene (*IL-1B*) promoter polymorphism rs16944 (-511, C > T) in cutaneous melanoma patients and healthy controls from Northeastern Italy, a region with a high incidence of melanoma. The CC genotype and C allele were more frequent in melanoma cases than in healthy controls. Among melanoma patients, the CC genotype was more frequent in Stage I disease and in melanomas with Breslow thickness ≤ 0.75 mm, but less frequent in Stage IV melanoma. The CT genotype was associated with approximately 3-fold higher odds of Stage IV disease and approximately 2- to 3-fold higher odds of lower-limb or lower-extremity melanoma, including after multivariable adjustment. Thus, rs16944 emerged as a possible candidate marker associated with melanoma susceptibility, Stage IV status, and lower-extremity localization. Independent studies are required to confirm these preliminary, exploratory findings.

## 1. Introduction

Cutaneous melanoma is the most aggressive and fatal skin cancer and shows an increasing incidence worldwide, particularly among White populations, with ethnic and geographical specificities [1,2]. In Northern Italy, the incidence of cutaneous melanoma is approximately twice that observed in Southern Italy [3,4]; in Italy overall, the estimated age-standardized incidence rates per 100,000 (ASR) for the year 2025 are 29.0 for men and 25.4 for women [3].

Inflammation, and particularly the inflammasome response, plays a major role in cutaneous melanoma [1,5,6,7]. This is also supported by recent innovative therapies aimed at restoring the antitumor immunosuppressive activities of T cells in the tumor microenvironment [8,9,10,11]. Modern immunotherapy has revolutionized the treatment of patients with melanoma; however, many patients still fail to respond to therapy or subsequently relapse [1,8,9,10,11,12]. Personalized approaches to cutaneous melanoma prevention, prognosis, and therapy remain a major challenge in current research and clinical management [1,9,12]. In this context, a number of genetic polymorphisms have been investigated for their association with cutaneous melanoma. In particular, polymorphisms of the vitamin D receptor (VDR) have been associated with an increased risk of cutaneous melanoma [13,14]. Genetic variants of the inflammasomes NLRP1 (nucleotide-binding domain, leucine-rich-containing family, pyrin domain-containing-1) rs12150220 and NLRP3 rs35829419, which are known to modulate levels of the pro-inflammatory cytokine interleukin (IL)-1β, have been associated with melanoma susceptibility, nodular melanoma, and fair skin among female patients [15]. Additionally, one study observed that gain-of-function mutations in NLRP1, the predominant inflammasome sensor protein in human skin keratinocytes, which is responsible for constitutive secretion of IL-1 by keratinocytes, increase paracrine IL-1 signaling, ultimately inducing skin inflammation, epidermal hyperplasia, and a predisposition to skin cancer [16].

Moreover, a variable number of tandem repeat (VNTR) polymorphism in the IL-1 receptor antagonist (IL-1RA) gene (*IL-1RN*), which encodes IL-1RA, a factor that antagonizes IL-1β activity, has been associated with increased cutaneous melanoma risk [17]. Interestingly, a very recent study showed that the *IL-1B* rs1143634 (+3954, C > T) polymorphism is associated with cutaneous melanoma susceptibility and upper-limb melanoma [18].

Several lines of evidence indicate a relationship between components of the IL-1 cytokine/receptor superfamily and cancer [19,20,21,22]. Specifically, the key pro-inflammatory cytokine IL-1β exerts multiple and sometimes opposing effects, including the promotion of angiogenesis and tumor invasion, which are associated with poor prognosis, as well as protective antitumor activities [19,20,21,22,23,24,25,26]. Therefore, single nucleotide polymorphisms (SNPs) in IL-1 family genes have been investigated in relation to cancer and melanoma [17,18,26,27,28,29].

The polymorphism in the promoter region of the *IL-1B* gene at position -511, rs16944, has been investigated in several cancers, including breast [30,31], prostate [32] and lung [33] cancer, with variable results. Few studies have examined the association between *IL-1B* promoter polymorphisms and skin cancers. One study found no association between the *IL-1B* polymorphism at position -598 T > C and cutaneous melanoma, although the same study found that the IL-1 decoy receptor type 2 (IL-1R2) rs4141134 GG genotype was more common in patients with the nodular subtype, and that the frequency of GG or GA genotypes was higher in patients with Clark levels III-V [34]. Furthermore, another study found that the *IL-1B* -511 TT genotype was associated with thinner invasive tumors at presentation, as assessed by Breslow thickness at the cut-off point of 1.5 mm, while no effects of the SNP were observed on susceptibility to or prognosis of cutaneous malignant melanoma [35].

In the present study, we investigated the association between the *IL-1B* rs16944 (-511 C > T) polymorphism and cutaneous melanoma susceptibility, as well as its relationship with clinical features, in an ethnically homogeneous Caucasian population of patients and healthy controls residing in the Friuli-Venezia Giulia (FVG) Region of Northeastern Italy.

## 2. Methods

### 2.1. Participants

Cutaneous melanoma patients and healthy controls were recruited at the Dermatology Clinic of the University Hospital of Udine [17,18], where routine diagnostic protocols were used to assess clinical status. The Institutional Ethics Committee of the University of Udine approved the study protocol, which complied with the Declaration of Helsinki. The study had an observational case–control design. All participants were alive at recruitment and signed written informed consent.

A total of 133 unrelated hospitalized patients or outpatients of both sexes, aged 30–90 years, with a documented diagnosis of cutaneous melanoma, participated in the study. The healthy control group included 900 asymptomatic healthy controls of both sexes (460 males and 440 females), aged 30–90 years, who were matched by age with melanoma patients. The larger control group reflected the availability of well-characterized age-matched controls from the same source population and was retained to maximize the precision of genotype-frequency estimates rather than being down-sampled. Study participants were recruited in a geographically restricted area of Northeastern Italy, within the Friuli-Venezia Giulia (FVG) Region, and the referral area was located within 100 km of the Udine University Hospital; therefore, major differences in broad regional environmental exposure were considered unlikely. None of the study participants was living or working in an area with high per- and polyfluoroalkyl substance (PFAS) water and soil contamination, such as that present in the nearby Veneto Region [36]. This criterion was considered important in view of the association between melanoma and environmental pollution [36,37,38]. Inclusion criteria for all study subjects were Caucasian Italian ethnicity, residence in the FVG Region, and at least two Italian grandparents born in the FVG Region or in the Austro-Hungarian territory before World War I, as previously described [13,14,17]. These ancestry-related criteria were based on self-reported ethnicity and family geographic origin; genome-wide ancestry markers and principal-component analyses were not available. Controls were recruited among healthy subjects attending the Dermatology Clinic for preventive check-up visits or through announcements at the University Hospital. Controls recruited through both routes were required to meet the same geographic and ancestry-related criteria and were intended to represent the same source population from which cases arose. A standardized dermatologist-performed whole-body examination was not a uniform inclusion requirement for all controls. None of the enrolled subjects was paid for participation in the study. Among healthy controls, 458 subjects had been previously studied for the rs16944 (-511) polymorphism [39,40]. Exclusion criteria for healthy controls included any lifetime history of malignant or benign tumors and major acute or chronic diseases, such as severe autoimmune diseases, including type 1 diabetes [14,17,18,41].

Cutaneous melanoma was diagnosed using immunohistological findings obtained after surgical excision of nevi with clinical and dermoscopic characteristics suggestive of malignancy. Classification of melanoma stages was performed according to the American Joint Committee on Cancer (AJCC) TNM staging framework, using the available clinical, histological, and radiological findings, as previously described [17,18,42]. Breslow thickness was additionally analyzed as a separate clinicopathological variable because it is a major prognostic feature of cutaneous melanoma and a component of AJCC T categorization; its separate presentation also permits direct comparison with our previous studies and prior literature. Inclusion criteria for melanoma cases included cutaneous melanoma only; mucosal melanomas were excluded. For patients with multiple melanomas, the major melanoma characteristics were included in the study analyses according to the histological assessment of the major primary tumor T grade [17,42]. For clarity, only the five most prevalent histologic subtypes were specifically indicated [43]. Tumor infiltrating lymphocytes (TILs) were defined as absent, non-Brisk, or Brisk according to the Clark et al. classification [44].

Non-metastatic melanoma patients (NMetM, Stage I and II) were defined as patients without metastasis after at least 5 years from initial melanoma diagnosis [17,18].

A structured questionnaire was used to collect data on demographic characteristics, medical history, family history of skin cancers, smoking habits, and lifestyle, as previously described [14,17,18]. Detailed melanoma risk-factor variables were available for all or nearly all cases but only a subset of controls; the variable-specific completeness and rationale for the main susceptibility model are described in the Statistical Analysis section. Phototype was assessed according to the Fitzpatrick criteria [45]. Body mass index (BMI) was calculated as weight in kilograms divided by height in meters squared (m^2^); BMI ≥ 30 kg/m^2^ was considered obese.

### 2.2. Genetic Analysis of IL-1B rs16944 (-511) Polymorphism

The *IL-1B* rs16944 polymorphism was determined by DNA restriction fragment analysis after PCR amplification with appropriate primers (PCR-RFLP), following extraction of genomic DNA from venous blood samples, as previously described [39,40]. The same validated PCR-based restriction-fragment protocol and genotype-scoring criteria were applied to all samples, and all genetic analyses were performed by personnel blinded to the demographic and clinical data of the recruited subjects.

### 2.3. Statistical Analysis

Continuous variables were expressed as the mean ± standard deviation (SD), and Student’s *t* test for independent samples was used for between-group comparisons. Tests for deviations from Hardy–Weinberg equilibrium (HWE) were performed as previously described [14], using a chi-square test for the distribution of genetic variables in cutaneous melanoma patients and, separately, in healthy controls. Odds ratios (ORs) and 95% confidence intervals (CIs) were calculated for categorical variables, and *p* values for two-sided Pearson’s chi-squared or Fisher’s exact test were reported as appropriate.

Multivariable logistic regression analysis was performed among melanoma patients to assess whether the associations between *IL-1B* rs16944 genotypes and selected clinicopathological features were independent of sex and age at first melanoma diagnosis. Separately, sex-adjusted binary logistic regression was performed for case–control comparisons of rs16944 genotypes and melanoma susceptibility, including all 133 cases and 900 controls. Adjusted odds ratios (aORs) and 95% confidence intervals (CIs) were calculated using binary logistic regression models. Established melanoma risk factors such as phototype, nevus count, family history of melanoma, UV exposure, and indoor tanning were not included in the main case–control susceptibility model because, depending on the variable, data were available for all or nearly all cases but only 154–157 of 900 controls; complete-case adjustment would therefore have substantially reduced the control sample and could have introduced selection bias. No single genetic inheritance model was prespecified. The genotype contrasts examined (CC versus TT + CT, TT + CT versus CC, and CT versus TT + CC) were treated as exploratory comparisons rather than as a confirmatory model-selection procedure. For allele-level analyses among melanoma patients, additive allele-dosage models were used. Additional exploratory sensitivity models were performed for lower-extremity localization by further adjusting for superficial spreading melanoma, acral lentiginous melanoma, and tumor-infiltrating lymphocyte absence.

A two-sided *p* value ≤ 0.050 was considered statistically significant, and a *p* value ≤ 0.100 was evaluated as a trend and highlighted by ^. No correction for multiple testing was applied because analyses beyond the main case–control susceptibility analysis were exploratory and hypothesis-generating; nominal *p* values should therefore not be interpreted as confirmatory evidence. A sensitivity power calculation for the main CC versus TT + CT case–control comparison used the realized sample sizes (133 cases and 900 controls), the CC frequency among controls (44.4%), a two-sided α = 0.05, and the normal approximation for two independent proportions. Statistical analyses were performed using SPSS for Windows, version 28 (SPSS Inc., Chicago, IL, USA).

## 3. Results

A total of 1033 subjects were genotyped for the rs16944 polymorphism located in the *IL-1B* gene promoter at position -511. The mean age of melanoma patients was 60.90 ± 12.84 years and did not differ from that of healthy controls, which was 61.89 ± 16.47 years (*p* = 0.474). Cases and controls did not differ for male sex (75/133 versus 460/900, OR = 1.24, CI = 0.86–1.78, *p* = 0.256). As a genotyping quality-control assessment, genotype distributions were in Hardy–Weinberg equilibrium (HWE) in both melanoma patients (χ^2^ = 0.184; *p* = 0.668) and healthy controls (χ^2^ = 0.158; *p* = 0.691).

Table 1 shows the distribution of *IL-1B* rs16944 genotypes and alleles. Among healthy controls, genotype frequencies were TT 11.6%, CT 44.0%, and CC 44.4%; among cutaneous melanoma patients, they were TT 6.0%, CT 39.8%, and CC 54.1%. The TT genotype showed a protective trend (OR = 0.49, *p* = 0.060 ^), whereas the frequency of the CT genotype did not differ between groups (OR = 0.84, *p* = 0.368). In contrast, the CC genotype was associated with modestly higher odds of melanoma (OR = 1.48, *p* = 0.037). In sex-adjusted case–control models including all 133 cases and 900 controls, the CC genotype remained associated with higher odds of melanoma (aOR = 1.46, CI = 1.01–2.11, *p* = 0.042), whereas the CT genotype was not associated (aOR = 0.84, CI = 0.58–1.22, *p* = 0.357) and the TT genotype showed a protective trend (aOR = 0.51, CI = 0.24–1.07, *p* = 0.073 ^). In an additive allele-dosage model, each additional C allele was associated with higher odds of melanoma (aOR = 1.42, CI = 1.06–1.91, *p* = 0.018). The sensitivity power calculation indicated 80% power to detect ORs of at least 1.69 or at most 0.58 for the main CC versus TT + CT comparison; smaller effects were less well powered. Consistently, the T allele was protective (OR = 0.69, *p* = 0.014), while the C allele was associated with higher odds of melanoma (OR = 1.44, *p* = 0.014), as shown in Table 1.

Table 2 compares 63 patients with metastatic melanoma (MetM, Stage II and IV) and 70 patients with non-metastatic melanoma (NMetM, Stage I and II). No statistically significant differences were observed between the two groups; however, the CT genotype tended to be more frequent in MetM than in NMetM patients (47.6% vs. 32.9%; OR = 1.86, *p* = 0.084 ^). When allele frequencies were compared in MetM versus NMetM patients, no statistically significant differences were observed.

Furthermore, as shown in Table 2, MetM patients did not differ from healthy controls. In contrast, the CC genotype and C allele were more frequent in NMetM patients than in healthy subjects (OR = 1.87, *p* = 0.013; and OR = 1.64, *p* = 0.016, respectively).

Table 3 reports the comparison between the CC and TT + CT genotypes among melanoma patients according to demographic and clinical characteristics.

Notably, the CC genotype was more frequent in Stage I melanoma (OR = 2.45, *p* = 0.016) and less frequent in Stage IV melanoma (OR = 0.37, *p* = 0.029) compared with the TT + CT genotypes. The CC genotype was more frequent in upper-limb melanomas (9.7% vs. 3.3%), although this difference was not statistically significant (*p* = 0.159). In contrast, the CC genotype was less frequent in lower-limb melanomas (13.9% vs. 29.5%, OR = 0.39, *p* = 0.031) and in melanomas with ≥1 mitosis/mm^2^ (52.8% vs. 76.3%, OR = 0.35, *p* = 0.006). Furthermore, the CC genotype was more frequent in melanomas with Breslow thickness ≤0.75 mm (33.3% vs. 18.0%, OR = 2.27, *p* = 0.049). CC carriers were less likely to have additional non-melanoma skin cancer (8.3% vs. 26.2%, OR = 0.26, *p* = 0.008) than TT + CT carriers; specifically, among CC carriers, 6 patients had basal-cell carcinomas, whereas among TT + CT carriers, 11 had basal-cell carcinomas, 1 patient had squamous-cell carcinoma, and 4 patients had squamous-cell carcinomas plus basal-cell carcinomas. Notably, all five patients with a squamous-cell carcinoma in addition to melanoma carried the CT genotype; however, because of the very small number of cases, no separate statistical analysis was performed for this subgroup.

Consistently, among melanoma patients, the TT + CT genotypes, compared with the CC genotype, were less likely to be associated with a Stage I disease (OR = 0.41, CI = 0.20–0.84, *p* = 0.016), and Breslow thickness ≤0.75 mm (OR = 0.44, CI = 0.19–1.00, *p* = 0.049). Furthermore, the TT + CT genotypes, compared with the CC genotype, were more likely to be associated with Stage IV disease (OR = 2.70, CI = 1.10–6.62, *p* = 0.029), lower-limb localization (OR = 2.60, CI = 1.09–6.17, *p* = 0.031), ≥1 mitosis/mm^2^ (OR = 2.88, CI = 1.35–6.13, *p* = 0.006), and additional non-melanoma skin cancer (OR = 3.91, CI = 1.42–10.8, *p* = 0.008).

When comparing the heterozygous CT genotype (n = 53) versus the homozygous TT + CC genotypes (n = 80) among melanoma patients, the only statistically significant findings were found for Stage I disease (26.4% vs. 47.5% OR = 0.40, CI = 0.19–0.84, *p* = 0.016), Stage IV melanoma (30.2% vs. 12.5% OR = 3.03, CI = 1.25–7.33, *p* = 0.014), lower-limb localization (30.2% vs. 15.0%, OR = 2.45, CI = 1.05–5.73, *p* = 0.038), and additional non-melanoma skin cancer (30.2% vs. 7.5%, OR = 5.33, CI = 1.93–14.8, *p* = 0.001). Moreover, a trend was observed for MetM (56.6% vs. 41.2%, OR = 1.86, CI = 0.92–3.75, *p* = 0.084 ^), and for ≥1 mitosis/mm^2^ (71.7% vs. 56.2%, OR = 1.97, CI = 0.94–4.14, *p* = 0.074 ^). No other trends or statistically significant differences were observed for the demographic and clinical characteristics examined in Table 3.

To determine whether the main genotype-associated clinical findings were independent of sex and age at first melanoma diagnosis, we performed multivariable logistic regression analyses among melanoma patients. The CC genotype remained independently associated with Stage I melanoma (aOR = 2.49, CI = 1.20–5.18, *p* = 0.015) and with Breslow thickness ≤ 0.75 mm (aOR = 2.29, CI = 1.01–5.18, *p* = 0.048). Conversely, the CC genotype remained inversely associated with Stage IV melanoma (aOR = 0.37, CI = 0.15–0.90, *p* = 0.029). The CT genotype remained significantly associated with Stage IV melanoma (aOR = 3.09, CI = 1.27–7.54, *p* = 0.013), lower-limb localization (aOR = 2.51, CI = 1.05–5.96, *p* = 0.038), and lower-extremity localization, defined as lower limbs plus feet (aOR = 3.32, CI = 1.45–7.62, *p* = 0.005). The TT + CT genotypes also remained associated with Stage IV melanoma (aOR = 2.72, CI = 1.11–6.66, *p* = 0.029), lower-limb localization (aOR = 2.62, CI = 1.09–6.30, *p* = 0.032), lower-extremity localization (aOR = 3.13, CI = 1.36–7.19, *p* = 0.007), and ≥1 mitosis/mm^2^ (aOR = 2.88, CI = 1.35–6.16, *p* = 0.006). The CT genotype was also independently associated with ≥1 mitosis/mm^2^ (aOR = 2.28, CI = 1.05–4.92, *p* = 0.037) and additional non-melanoma skin cancer (aOR = 5.39, CI = 1.90–15.26, *p* = 0.002), whereas the association with metastatic melanoma showed only a trend after adjustment (aOR = 1.87, CI = 0.92–3.80, *p* = 0.082 ^).

Demographic and lifestyle characteristics did not differ between groups.

Table 4 illustrates genotype and allele frequencies in Stage IV melanoma patients (n = 26) compared with melanoma patients in other Stages (n = 107) and with healthy controls. Among melanoma patients, the CT and TT + CT genotypes were associated with an increased risk of Stage IV melanoma (OR = 3.03, *p* = 0.014; aOR = 3.09, CI = 1.27–7.54, *p* = 0.013, and OR = 2.70, *p* = 0.029; aOR = 2.72, CI = 1.11–6.66, *p* = 0.029, respectively); consequently, the CC genotype was protective (OR = 0.37, *p* = 0.029; aOR = 0.37, CI = 0.15–0.90, *p* = 0.029). No significant findings were observed for the T and C alleles. Percentages of females did not differ between the two groups of melanoma patients (42.3% vs. 43.9%, OR = 0.94, *p* = 0.881).

When Stage IV melanoma patients were compared with healthy controls, the CT genotype showed a trend toward a higher frequency (OR = 2.04, *p* = 0.082 ^); no other data were statistically significant.

Table 5 shows genotype and allele frequencies in patients with melanoma localized in the lower limbs (n = 28) compared with the other melanoma patients (n = 107) and with healthy controls.

The CT and TT + CT genotypes were more frequent in melanoma localized in the lower limbs than in melanoma at other anatomical sites (OR = 2.45, *p* = 0.038; aOR = 2.51, CI = 1.05–5.96, *p* = 0.038, and OR = 2.60, *p* = 0.031; aOR = 2.62, CI = 1.09–6.30, *p* = 0.032, respectively); conversely, the CC genotype was less frequent (OR = 0.39, *p* = 0.031; aOR = 0.38, CI = 0.16–0.92, *p* = 0.032). In additive allele-dosage models, the T allele showed a trend toward association with lower-limb localization after adjustment for sex and age at first melanoma diagnosis (aOR = 1.90, CI = 0.96–3.74, *p* = 0.064 ^), whereas the C allele showed the reciprocal trend (aOR = 0.53, CI = 0.27–1.04, *p* = 0.064 ^).

Among lower-limb melanoma patients, 57.1% were females, compared with 40.0% of females with melanoma at other anatomical sites; however, this difference was not statistically significant (OR = 2.00, CI = 0.86–4.65, *p* = 0.108). The two groups of patients did not differ in any other demographic or clinical features, although TIL absence was found more frequently in lower-limb melanoma patients (46.4% vs. 30.5%, OR = 1.98, CI = 0.84–4.63, *p* = 0.116).

No differences in genotype and allele frequencies were noted between patients with melanoma in the lower limbs and healthy controls.

Table 6 shows the comparison of genotype and allele distributions between patients with melanoma localized in the lower extremities (n = 35), defined as lower limbs plus feet, and those with melanoma at other anatomical sites (n = 98), and healthy controls. The TT genotype did not differ between groups; the CT and TT + CT genotypes were associated with an increased risk (OR = 3.09, *p* = 0.005, and OR = 3.03, *p* = 0.007, respectively); conversely, the CC genotype was protective (OR = 0.33, *p* = 0.007). The T allele was associated with an increased risk (OR = 1.92, *p* = 0.031), while the C allele was protective (OR = 0.52, *p* = 0.031).

The association of the CT genotype and TT + CT genotypes with lower-extremity melanoma localization remained significant after adjustment for sex and age at first melanoma diagnosis (aOR = 3.32, CI = 1.45–7.62, *p* = 0.005, and aOR = 3.13, CI = 1.36–7.19, *p* = 0.007, respectively). Conversely, the CC genotype remained inversely associated with lower-extremity localization (aOR = 0.32, CI = 0.14–0.73, *p* = 0.007). In additive allele-dosage models, the T allele remained associated with lower-extremity localization (aOR = 2.02, CI = 1.06–3.85, *p* = 0.033), whereas the C allele showed the reciprocal inverse association (aOR = 0.50, CI = 0.26–0.94, *p* = 0.033).

When lower-extremity melanoma patients were compared with healthy controls, the CT genotype showed a trend toward a higher frequency (OR = 1.91, *p* = 0.066 ^); no other comparisons were statistically significant.

Furthermore, Table 7 illustrates the demographic and clinical characteristics of patients with melanoma localized in the lower extremities compared with those of the remaining melanoma patients. Patients with lower-extremity melanoma were more likely to be female (OR = 2.47, *p* = 0.025), to have acral lentiginous melanoma (OR = 12.5, *p* = 0.026), and to show an absence of tumor-infiltrating lymphocytes (TILs) (OR = 2.29, *p* = 0.041). Conversely, they were less likely to be male (OR = 0.40, *p* = 0.025) and to have superficial spreading melanoma (OR = 0.42, *p* = 0.035). Finally, they showed a trend toward a higher frequency of ≥1 mitosis/mm^2^ (OR = 2.41, *p* = 0.052 ^). Demographic and lifestyle characteristics did not differ between groups.

Based on these findings, additional exploratory sensitivity models were performed for lower-extremity localization by further adjusting for sex, age at first melanoma diagnosis, superficial spreading melanoma, acral lentiginous melanoma, and TIL absence. In these fully adjusted models, the association between the CT genotype and lower-extremity localization remained significant (aOR = 3.50, CI = 1.44–8.50, *p* = 0.006). Similarly, the TT + CT genotypes remained associated with lower-extremity localization (aOR = 3.20, CI = 1.32–7.75, *p* = 0.010), whereas the CC genotype remained inversely associated with this localization (aOR = 0.31, CI = 0.13–0.75, *p* = 0.010). In additive allele-dosage models, the T allele remained associated with lower-extremity localization (aOR = 1.98, CI = 1.01–3.89, *p* = 0.048), whereas the C allele showed the reciprocal inverse association (aOR = 0.51, CI = 0.26–0.99, *p* = 0.048).

Table 8 reports the comparison of genotype and allele distributions between patients with melanoma localized in the upper extremities (n = 10), defined as upper limbs plus hands, and those with melanoma at other anatomical sites (n = 123). No statistically significant differences were observed among melanoma patients, although the CC genotype and C allele were more frequent in patients with melanoma localized in the upper extremities than in those with melanoma at other anatomical sites (OR = 3.69, *p* = 0.108, and OR = 3.37, *p* = 0.110, respectively). In the small subgroup of 10 upper-extremity melanoma cases, the CC genotype and C allele were also nominally more frequent than in healthy controls (OR = 5.00, *p* = 0.042, and OR = 4.55, *p* = 0.043, respectively). Given the very small number of cases and the resulting imprecision of the estimates, these findings are preliminary and should be interpreted cautiously.

Overall, these findings suggest that the association between the *IL-1B* rs16944 polymorphism and melanoma localization in the extremities differs according to anatomical site, with opposite patterns observed for upper and lower extremities.

## 4. Discussion

In the present study, the genotype distribution of the rs16944 polymorphism among 900 Caucasian healthy controls was TT 11.6%, CT 44.0%, and CC 44.4%. These data are broadly similar to those of a previous investigation conducted in 458 healthy subjects from Northern Italy, in which the frequencies were TT 9.4%, CT 45.9%, and CC 44.8% [40], as well as to those reported in other Italian studies [46] conducted in Northern Italy, where frequencies were TT 13.0%, CT 45.8%, and CC 41.2%, and in Southern Italy, where frequencies were TT 9.6%, CT 43.8%, and CC 46.6%. A meta-analysis [47] reported frequencies in European populations ranging from 7.0% to 19.4% for TT, 20.1% to 57.5% for CT, and 30.0% to 66.0% for CC. A U.S. study performed in 452 Caucasian individuals undergoing coronary angiography found the following frequencies: TT 12.2%, CT 42.7%, and CC 45.1% [48]. Allele frequencies for the T and C alleles were reported to be, respectively: 33.2% and 66.8% in European populations, 53.6% and 46.4% in African Americans, 46.4% and 53.6% for East Asians, and 61.0% and 39% for South Asians, indicating substantial ethnic differences in rs16944 distribution [49].

Such differences may partly explain the variable associations reported between this polymorphism and disease across populations. For example, according to a meta-analysis study, a statistically significant increased risk of gastric cancer associated with the *IL-1B* rs16944 (-511) SNP was observed in Caucasian, but not in Asian, populations [50]. Variable results have also been reported for associations between the rs16944 polymorphism and breast and cervical cancer, for which the associations were stronger in Asian than Caucasian populations [30,51]. Therefore, population background should be carefully considered when interpreting the biological and clinical impact of this SNP.

The rs16944 polymorphism is located in the promoter region of the *IL-1B* gene and may influence IL-1β expression. Some prior studies reported reduced IL-1β production or mRNA expression in carriers of the T allele or TT genotype and relatively higher IL-1β-related activity in CC carriers [52,53,54]. However, the literature is not fully consistent, and functional consequences may depend on tissue type, inflammatory context, environmental exposure, and interactions with other immune-regulatory variants [48]. We did not measure IL-1β expression, cytokine levels, or activity in this cohort; therefore, no functional effect of the CC or CT genotype can be inferred from our data.

In the present study, the CC genotype and C allele were associated with modestly higher odds of cutaneous melanoma (OR = 1.48 and OR = 1.44, respectively), indicating a modest association in this cohort rather than establishing causality. The association was stronger among patients with non-metastatic melanoma, in whom the CC genotype and C allele were more frequent than in healthy controls (OR = 1.87 and OR = 1.64, respectively). Two previous studies reported associations of *IL-1RN* rs2234663 and *IL-1B* rs1143634 variants (both considered to increase IL-1β activity) with melanoma susceptibility, without differences between metastatic and non-metastatic melanoma patients [17,18].

Among melanoma patients, the CC genotype was associated with Stage I disease (OR = 2.45, aOR = 2.49) and Breslow thickness ≤ 0.75 mm (OR = 2.27, aOR = 2.29), whereas it was less frequent in Stage IV melanoma. These cross-sectional differences in genotype frequency do not establish disease trajectory, progression, or causality. One possible but untested hypothesis is that IL-1β-related increased inflammatory activity may have context-dependent associations with early localized disease; direct functional studies are required because IL-1β was not measured in this cohort [22,25,26].

Conversely, the CT genotype was associated with Stage IV disease (OR = 3.03; aOR = 3.09). The CT genotype should not be interpreted as having a defined intermediate functional effect on IL-1β production, because genotype-function relationships may vary by biological context and IL-1β expression was not measured in this study. Accordingly, the Stage IV association should be viewed as an exploratory association with prevalent disease status rather than evidence that the CT genotype drives melanoma progression by reduced IL-1β activity associated with the T allele [52,53,54].

Of note, melanoma patients with different rs16944 genotypes did not differ in demographic and clinical/lifestyle characteristics including age at first melanoma diagnosis, sex, smoking and coffee habits, sunburns, number of nevi, fair skin, and indoor tanning.

IL-1β has a distinctive role in innate immune activation and trained immunity [19,26,27,39,40,52,55], but these general biological observations do not establish the functional consequence of rs16944 in melanoma.

Early-stage melanoma can precede invasive disease, but not all lesions follow the same clinical trajectory, and host immune responses may contribute to this heterogeneity [56,57].

Experimental studies illustrate the context dependence of IL-1β in melanoma: IL-1β blockade increased lung metastasis in one murine model, whereas another antitumor intervention increased IL-1β together with intratumoral immune activation [58,59]. These findings provide biological context but do not demonstrate the mechanism underlying the associations observed in our cohort.

Among melanoma patients, the heterozygous CT genotype and the TT + CT genotypes were associated with approximately 3-fold higher odds of prevalent Stage IV melanoma. Because the study is cross-sectional and the Stage IV subgroup included only 26 patients, these results do not establish that rs16944 drives progression or metastatic dissemination and should be considered exploratory.

A single genetic variant is unlikely to be the sole direct cause of melanoma onset or metastatic disease, and our observational data cannot establish a mechanistic role. The observed associations instead motivate direct functional studies and replication in independent cohorts.

The different frequencies of the CC genotype in Stage I and Stage IV melanoma could be compatible with the context-dependent roles of IL-1β in inflammation and antitumor immunity [18,19,20,22,23,26], but our data do not distinguish biological effects from sampling variability or other unmeasured factors.

The higher frequency of the rs16944 CC genotype in Stage I disease and its lower frequency in Stage IV melanoma reflect a cross-sectional association between genotype and disease stage. They do not establish that the CC genotype promotes melanoma initiation, prevents progression, or determines disease trajectory. Interactions with other *IL-1B* variants, other immune-regulatory genes, and environmental factors remain possible and warrant investigation.

Another noteworthy finding of our study was the association between the rs16944 SNP and melanoma localization in the extremities. The CT genotype and T allele were associated with melanoma localized in the lower extremities, whereas the CC genotype and C allele showed an opposite pattern and were more frequent in patients with upper-extremity melanoma than in healthy controls. In this study, “lower-extremity melanoma” denotes an anatomical site (lower limb plus foot) and is not synonymous with acral lentiginous melanoma, which was analyzed separately as a histological subtype.

Overall, these exploratory findings suggest that the relationship between rs16944 and melanoma anatomical localization may differ according to body site. To our knowledge, this is one of the first preliminary observations of an association between a genetic polymorphism and lower-extremity melanoma localization.

The biological explanation for this finding requires further investigation. Several studies have examined risk factors for melanoma localization in the lower limbs and extremities; the main causal relationship is considered to be high sun exposure and tanning [60,61]; however, causal relationships have so far remained elusive [43,60]. Melanoma arising in different anatomical sites may differ in terms of ultraviolet exposure patterns, local immune surveillance including inflammasomes, skin structure, vascularization, lymphatic drainage, and microenvironmental characteristics [5,16,62,63,64,65,66].

Consolidated evidence in many epidemiological settings has shown that lower-limb melanomas are more frequent in women [43,60,61,67]. Consistently, in our study, patients with melanoma localized in the lower extremities were more likely to be female. They were also more likely to have acral lentiginous melanoma and an absence of tumor-infiltrating lymphocytes, whereas superficial spreading melanoma was less frequent. We did not observe a higher frequency of nevi, fair skin, indoor tanning use or other lifestyles in patients with lower-limb melanoma than in those with melanoma localized in other areas.

A U.S. study that analyzed all skin melanoma cases between 2000 and 2019 in which 50,109 patients, 92.4% of whom were White, were diagnosed with melanoma in the lower limbs and hips, observed that more women (70.8%) and younger people (55.2 ± 16.5 years) were affected by lower-extremity melanoma, with better survival rates (87.5% at 5 years) than melanomas in other skin regions; additionally, most of them were diagnosed at localized Stage I and II (83.7%) [43]. In the U.S. epidemiologic survey, the most represented histologic subtypes were superficial spreading (35.7%), nodular (6.6%), acral lentiginous (4.3%), and lentigo maligna (1.5%) melanomas; however, 49.2% were classified as “not otherwise specified”. Such American percentages are roughly in line with our findings about histologic subtype distribution in lower-extremity melanoma patients, with the exception of the nodular subtype, which was 32.8% in our cohort.

Taken together, these features may indicate a distinct clinicopathologic profile in lower-extremity melanoma. The observed association with rs16944 may reflect complex interactions among sex, histologic subtype, immune infiltration, environmental exposure, and anatomical site rather than an effect of rs16944 alone. This exploratory observation requires independent replication and functional testing.

Interestingly, however, in our cohort, patients with lower-extremity melanoma did not differ from those with other site melanoma localizations in regard to demographic and clinical/lifestyle characteristics including age at first melanoma diagnosis, BMI, smoking and coffee habits, sunburns, number of nevi, fair skin, and indoor tanning.

Indeed, the association of the rs16944 CT genotype with lower-extremity melanoma remained statistically significant after adjustment for sex and age at first melanoma diagnosis (aOR = 3.32, *p* = 0.005), indicating that the observed association was not driven by female sex alone. Importantly, this association also remained significant in exploratory sensitivity models further adjusted for superficial spreading melanoma, acral lentiginous melanoma, and TIL absence (aOR = 3.50, *p* = 0.006). These findings suggest that the relationship between rs16944 and lower-extremity localization is not explained solely by sex distribution or by the main clinicopathological features that differed between lower-extremity and other-site melanomas. Nevertheless, because of the limited sample size and the small number of lower-extremity melanoma cases, this result should be interpreted as hypothesis-generating.

Sex-related differences in melanoma and the immunomodulatory effects of estrogens may be relevant to anatomical-site heterogeneity. Estrogen receptor β signaling has been linked experimentally to IL-1β production and antitumor immune responses in melanoma models [68,69,70,71]. These observations provide context for future mechanistic studies but do not directly explain the genotype associations reported here.

TIL absence was more frequent in lower-extremity melanoma in our cohort. Because TILs reflect host immune responses and have prognostic and therapeutic relevance [12,72], this observation may warrant further study. However, linking TIL absence to rs16944-dependent IL-1β activity would be speculative because IL-1β expression and tumor immune programs were not measured; confirmation will require larger cohorts with molecular characterization of the tumor microenvironment [52,53,54].

In our study, the upper-extremity subgroup comprised only 10 melanoma cases. The CC genotype and C allele showed nominally approximately 5-fold higher odds relative to healthy controls, but these estimates are based on very small numbers and should be regarded as preliminary. Another *IL-1B* polymorphism, rs1143634, has recently been associated with upper-limb melanoma [18].

Previous epidemiologic and molecular studies also support heterogeneity of melanoma by anatomical site, including sex differences, variation in associations with UV exposure and indoor tanning, and site-specific oncogenic features [66,73,74,75].

### 4.1. Limitations and Strengths

This study has several limitations. First, although the control group was large, only 133 melanoma patients were included, and several subgroup analyses involved small numbers. A sensitivity calculation for the main CC versus TT + CT case–control comparison showed 80% power only for ORs of at least 1.69 or at most 0.58; subgroup estimates were less well powered and may be unstable. Second, no correction for multiple testing was applied, and all secondary analyses should therefore be considered exploratory and hypothesis-generating. Third, the observational case–control design does not allow causal inference or reconstruction of disease trajectory. Fourth, functional assays were not included; therefore, the biological effect of rs16944 on IL-1β expression or activity in melanoma remains unknown. Fifth, established melanoma risk factors such as phototype, nevus count, family history, UV exposure, and indoor tanning were not available uniformly across the full control cohort and could not be incorporated into the main susceptibility model without a major reduction in sample size; residual confounding cannot therefore be excluded. Sixth, a standardized dermatologist-performed whole-body examination was not a uniform inclusion requirement for all controls. Seventh, possible interactions with other genetic variants, environmental exposures, and immune-related biomarkers were not fully explored. Eighth, ancestry was defined by self-reported ethnicity and regional family background, whereas genome-wide ancestry markers or principal-component analyses were not available; therefore, residual population stratification cannot be fully excluded. Ninth, although genotyping quality was supported by a uniform validated assay, blinded procedures, and Hardy–Weinberg equilibrium in both study groups, a dedicated prospective batch variable was not available for formal batch-effect modeling. Moreover, several multivariable logistic regression models were fitted in relatively small subgroups, such as Stage IV melanoma (n = 26) and lower-extremity melanoma (n = 35), with adjustment for multiple covariates. Finally, the study did not assess downstream gene-expression programs associated with rs16944. The association between non-melanoma skin cancers and rs16944 also requires independent assessment; the observation that all five melanoma patients with additional squamous-cell carcinoma carried the CT genotype was descriptive only and could not be statistically evaluated. For these reasons, the present results should be considered preliminary and hypothesis-generating.

Strengths of the present study include a well-defined study population; cases and controls were recruited in a restricted Italian geographical area using the same ancestry-related inclusion criteria, which helps reduce major differences in population background and broad regional environmental exposures. To our knowledge, this is the largest investigation of rs16944 genotype distribution in the Italian population. Furthermore, cutaneous melanoma patient information included not only melanoma stages and histologic features, but also melanoma localization and demographic/lifestyle characteristics.

### 4.2. Clinical Significance

This study provides preliminary evidence of an association between the *IL-1B* rs16944 (-511 C > T) polymorphism and melanoma susceptibility, disease stage, and anatomical localization. In this Northeast Italian cohort, CC genotype carriage was associated with modestly higher odds of melanoma and was more frequent in Stage I disease but less frequent in Stage IV disease. Conversely, the CT genotype was associated with prevalent Stage IV disease among melanoma patients. Another exploratory observation was the association of rs16944 with lower-limb/lower-extremity melanoma, for which CT and TT + CT genotypes showed approximately 3-fold higher odds among melanoma patients.

Overall, our findings indicate context-dependent associations between rs16944 and melanoma susceptibility, disease stage, and anatomical site; they do not establish a causal role of IL-1β-related genetic background. After independent replication, rs16944 variants may warrant evaluation as components of multifactorial melanoma risk models.

Future studies should investigate the relationship between the *IL-1B* rs16944 polymorphism and cutaneous melanoma in larger independent cohorts, ideally including different ethnic groups and geographically distinct populations. Integrated approaches may improve understanding of IL-1β-mediated effects in melanoma, also informing therapeutic strategies involving IL-1β inhibition. Our present study contributes to the debate on therapeutic approaches including IL-1β inhibition in cancer treatments [5,12,19,20,21,22,24,26,55,76,77].

Our results may open the way to functional studies aimed at determining whether the rs16944 polymorphism directly affects IL-1β expression in skin, melanoma tissue, or immune cells. In addition, it would be useful to evaluate interactions between the rs16944 and other polymorphisms, including variants in other regions of the *IL-1B* gene, as well as genes encoding other cytokines and receptors of the IL-1 family.

## 5. Conclusions

To our knowledge, this is the first report exploring the association between the *IL-1B* rs16944 polymorphism and cutaneous melanoma susceptibility, stage, and lower-extremity localization. These findings add preliminary information on a possible relationship between innate-immunity-related genetic variation and the clinical heterogeneity of cutaneous melanoma.

Our study showed that the *IL-1B* rs16944 (-511 C > T) polymorphism is associated with cutaneous melanoma susceptibility and selected clinical features in a Northern Italian population. The CC genotype was associated with modestly higher odds of melanoma and was more frequent in early-stage disease, whereas the CT genotype was associated with prevalent Stage IV melanoma and lower-limb/lower-extremity localization. These secondary associations are exploratory and do not establish progression, disease trajectory, or causality. The divergent patterns could be compatible with context-dependent IL-1β biology, but the functional mechanism remains untested. Further studies are required to confirm these observations and directly assess the biological mechanisms linking rs16944 to melanoma susceptibility and clinical phenotype.

Advances in precision personalized approaches to melanoma patients are needed because, although modern innovative immune therapies have a substantial beneficial impact on the survival of cutaneous melanoma patients, a considerable number of patients with metastatic melanoma still experience fatal outcomes, and more focused and efficient treatments are warranted [1,11,12,78].

Additional new biomarkers are needed to better identify high-risk patients at early stages, to optimize monitoring and possibly also to avoid overtreatment of patients with immune checkpoint inhibitors, thereby preventing potential toxicity caused by these therapies [12,78]. A better comprehension of the individual genetic background [79] of innate immunity may improve primary prevention strategies, allowing early detection and potentially contributing to the development of improved treatments and individualized therapy options, thereby enhancing patient outcomes.

## Figures and Tables

**Table 1:** Genotype and allele frequencies of the *IL-1B* rs16944 (-511) polymorphism in cutaneous melanoma patients (n = 133) compared with healthy controls (n = 900).

	Melanoma Patients (n = 133)	Healthy Controls (n = 900)	OR (CI), *p* Value, Melanoma vs. Healthy Subjects
*IL-1B* genotype			
TT	8 (6.0%)	104 (11.6%)	0.49 (0.23–1.03), 0.060 ^
CT	53 (39.8%)	396 (44.0%)	0.84 (0.58–1.22), 0.368
CC	72 (54.1%)	400 (44.4%)	**1.48 (1.02–2.13), 0.037**
TT + CT	61 (45.9%)	500 (55.6%)	**0.68 (0.47–0.98), 0.037**
*IL-1B* allele			
Allele T	69/266 (25.9%)	604/1800 (33.6%)	**0.69 (0.52–0.93), 0.014**
Allele C	197/266 (74.1%)	1196/1800 (66.4%)	**1.44 (1.08–1.93), 0.014**

Differences between groups were evaluated by OR and CI for categorical variables. Statistically significant differences were indicated in bold. Trends were denoted with ^.

**Table 2:** Genotype and allele frequencies of *IL-1B* rs16944 (-511) comparisons between metastatic melanoma (MetM) patients (n = 63), non-metastatic melanoma (NMetM) patients (n = 70), and healthy controls (n = 900).

	MetM (n = 63)	NMetM (n = 70)	OR (CI), *p* Value, MetM vs. NMetM	OR (CI), *p* Value, MetM vs. Healthy Controls (n = 900)	OR (CI), *p* Value, NMetM vs. Healthy Controls (n = 900)
*IL-1B* genotype					
TT	3 (4.8%)	5 (7.1%)	0.65 (0.15–2.84), 0.567	0.38 (0.12–1.24), 0.110	0.59 (0.23–1.50), 0.265
CT	30 (47.6%)	23 (32.9%)	1.86 (0.92–3.75), 0.084 ^	1.16 (0.69–1.93), 0.576	0.62 (0.37–1.04), 0.072 ^
CC	30 (47.6%)	42 (60.0%)	0.61 (0.30–1.21), 0.154	1.14 (0.68–1.90), 0.624	**1.87 (1.14–3.08), 0.013**
TT + CT	33 (52.4%)	28 (40.0%)	1.65 (0.83–3.28), 0.154	0.88 (0.53–1.47), 0.624	**0.53 (0.32–0.88), 0.013**
*IL-1B* allele					
Allele T	36/126 (28.6%)	33/140 (23.6%)	1.30 (0.75–2.25), 0.353	0.79 (0.53–1.18), 0.252	**0.61 (0.41–0.91), 0.016**
Allele C	90/126 (71.4%)	107/140 (76.4%)	0.77 (0.45–1.34), 0.353	1.26 (0.85–1.88), 0.252	**1.64 (1.10–2.45), 0.016**

Differences between groups were evaluated by OR and CI for categorical variables. Statistically significant differences were indicated in bold. Trends were denoted with ^.

**Table 3:** Comparison of clinical characteristics between the two genetic subgroups of *IL-1B* rs16944 (-511) polymorphism CC genotypes (n = 72) versus TT + CT genotypes (n = 61) among 133 melanoma patients.

Characteristic	CC Genotype (n = 72)	TT + CT Genotype (n = 61)	OR (CI), CC vs. TT + CT	*p* Value CC vs. TT + CT
Age < 50 years at study enrolment, n (%)	17 (23.6%)	12 (19.7%)	1.26 (0.55–2.90)	0.584
Age at study enrolment, years, mean ± SD	61.1 ± 13.4	60.6 ± 12.2	-	0.818
Age < 50 years at melanoma diagnosis	25 (34.7%)	23 (37.7%)	0.88 (0.43–1.79)	0.721
Age at melanoma diagnosis, years, mean ± SD	54.3 ± 13.9	53.8 ± 13.7	-	0.834
Females, n (%)	31 (43.1%)	27 (44.3%)	0.95 (0.48–1.89)	0.889
Males, n (%)	41 (56.9%)	34 (55.7%)	1.05 (0.53–2.09)	0.889
All grandparents born in FVG	49 (68.1%)	47 (77.0%)	0.63 (0.29–1.38)	0.250
BMI kg/m^2^, mean ± SD	25.5 ± 4.33	26.2 ± 3.49	-	0.285
BMI ≥30 kg/m^2^, n (%)	13 (18.1%)	9 (14.8%)	1.27 (0.50–3.22)	0.610
Present smoker, n (%)	5 (6.9%)	7 (11.5%)	0.58 (0.17–1.92)	0.368
Ever smoker, n (%)	33 (45.8%)	32 (52.5%)	0.77 (0.39–1.52)	0.447
≥ 20 years smoking ever in all subjects, n (%)	18 (25.0%)	22 (36.1%)	0.59 (0.28–1.25)	0.167
≥ 20 cigarettes/day ever in all subjects, n (%)	14 (19.4%)	11 (18.0%)	1.10 (0.46–2.63)	0.835
Cups of espresso coffee per day ≥ 2, n (%)	54 (75.0%)	46 (75.4%)	0.98 (0.44–2.16)	0.956
Phototype 1 and 2, n (%)	42 (58.3%)	35 (57.4%)	1.04 (0.52–2.07)	0.911
Nevi ≥ 50, n (%)	36 (50.0%)	30 (49.2%)	1.03 (0.52–2.04)	0.925
Low/no tanner, n (%)	43 (59.7%)	31 (50.8%)	1.43 (0.72–2.86)	0.304
Burns ≥ 5 lifelong, n (%)	37 (51.4%)	34 (55.7%)	0.84 (0.42–1.66)	0.616
Burns ≥ 10 lifelong, n (%)	24 (33.3%)	25 (41.0%)	0.72 (0.35–1.46)	0.363
Indoor tanning ≥1 ever, n (%)	16 (22.2%)	14 (23.0%)	0.96 (0.42–2.17)	0.920
MeM, n (%)	30 (41.7%)	33 (54.1%)	0.61 (0.30–1.21)	0.154
Stage I, n (%)	35 (48.6%)	17 (27.9%)	**2.45 (1.18–5.06)**	**0.016**
Stage II, n (%)	7 (9.7%)	11 (18.0%)	0.49 (0.18–1.35)	0.168
Stage III, n (%)	21 (29.2%)	16 (26.2%)	1.16 (0.54–2.49)	0.706
Stage IV, n (%)	9 (12.5%)	17 (27.9%)	**0.37 (0.15–0.90)**	**0.029**
Trunk, n (%)	44 (61.1%)	31 (50.8%)	1.52 (0.76–3.03)	0.234
Upper limb, n (%)	7 (9.7%)	2 (3.3%)	3.18 (0.63–15.9)	0.159
Lower limb, n (%)	10 (13.9%)	18 (29.5%)	**0.39 (0.16–0.92)**	**0.031**
Hands/feet, n (%)	3 (4.2%)	5 (8.2%)	0.49 (0.11–2.13)	0.339
Head/neck, n (%)	8 (11.1%)	5 (8.2%)	1.40 (0.43–4.53)	0.574
Superficial spreading, n (%)	40 (55.6%)	30 (49.2%)	1.29 (0.65–2.56)	0.463
Nodular, n (%)	25 (34.7%)	20 (32.8%)	1.09 (0.53–2.24)	0.814
Acral lentiginous, n (%)	2 (2.8%)	3 (4.9%)	0.55 (0.09–3.42)	0.523
Lentigo maligna, n (%)	1 (1.4%)	1 (1.6%)	0.85 (0.05–13.8)	0.906
Spitzoide, n (%)	2 (2.8%)	3 (4.9%)	0.55 (0.09–3.42)	0.523
Breslow thickness, mm, mean ± SD	1.86 ± 1.72	2.27 ± 2.00	-	0.201
Breslow thickness ≤ 0.75 mm, n (%)	24 (33.3%)	11 (18.0%)	**2.27 (1.00–5.14)**	**0.049**
Breslow thickness 0.76–1.00 mm, n (%)	7 (9.7%)	6 (9.8%)	0.99 (0.31–3.11)	0.982
Breslow thickness 1.01–2.00 mm, n (%)	18 (25.0%)	20 (32.8%)	0.68 (0.32–1.45)	0.323
Breslow thickness 2.01–4.00 mm, n (%)	13 (18.1%)	16 (26.2%)	0.62 (0.27–1.42)	0.257
Breslow thickness ≥ 4.01 mm, n (%)	10 (13.9%)	8 (13.1%)	1.07 (0.39–2.90)	0.896
Ulceration, n (%)	25 (34.7%)	26 (42.6%)	0.73 (0.36–1.48)	0.384
Mitosis ≥ 1 per mm^2^, n (%)	38 (52.8%)	45 (76.3%) ^b^	**0.35 (0.16–0.74)**	**0.006**
Regression, n (%)	13 (18.1%)	7 (11.9) ^b^	1.64 (0.61–4.41)	0.330
Brisk positive TILs ^a^, n (%)	20 (27.8%)	18 (30.5%) ^b^	0.88 (0.41–1.87)	0.732
Non-brisk TILs ^a^, n (%)	29 (40.3%)	18 (30.5%) ^b^	1.54 (0.74–3.18)	0.247
TILs ^a^ absence, n (%)	22 (30.6%)	23 (39.0%) ^b^	0.64 (0.31–1.32)	0.228
Microsatellitosis, n (%)	4 (5.6%)	1 (1.7%) ^b^	3.41 (0.37–31.4)	0.278
Epithelioid variant, n (%)	20 (27.8%)	17 (28.3%) ^c^	0.97 (0.45–2.09)	0.944
Fusate variant, n (%)	6 (8.3%)	7 (11.7%) ^c^	0.69 (0.22–2.17)	0.524
Small cell variant, n (%)	1 (1.4%)	1 (1.7%) ^c^	0.83 (0.05–13.6)	0.897
More than 1 melanoma, n (%)	11 (15.3%)	10 (16.4%)	0.92 (0.36–2.34)	0.860
Additional non-melanoma skin cancer, n (%)	6 (8.3%)	16 (26.2%)	**0.26 (0.09–0.70)**	**0.008**
Additional non-skin cancer, n (%)	16 (22.2%)	13 (21.3%)	1.05 (0.46–2.41)	0.899
Melanoma familiarity, n (%)	13 (18.1%)	5 (8.2%)	2.47 (0.83–7.37)	0.106
Dead ≤ 5 years from first diagnosis	9 (12.5%)	9 (14.8%)	0.83 (0.31–2.23)	0.705

Differences between groups were evaluated by OR and CI for categorical variables and by Student’s *t*-test for independent samples for continuous variables. ^a^ TILs, tumor infiltrating lymphocytes. ^b^ Data were available for 59 patients. ^c^ Data were available for 60 patients. Significant differences were indicated in bold.

**Table 4:** Genotype and allele frequencies of *IL-1B* rs16944 (-511) comparisons between Stage IV metastatic melanoma patients (n = 26), and other Stages melanoma patients (n = 107), and healthy controls (n = 900).

	Stage IV Melanoma Patients (n = 26)	Other Stages Melanoma Patients (n = 107)	OR (CI), *p* Value, Stage IV vs. Other Stages Melanoma Patients	OR (CI), *p* Value, Stage IV vs. Healthy Subjects (n = 900)
*IL-1B* genotype				
TT	1 (3.8%)	7 (6.5%)	0.57 (0.07–4.86), 0.608	0.31 (0.04–2.28), 0.248
CT	16 (61.5%)	37 (34.6%)	**3.03 (1.24–7.33), 0.014**	2.04 (0.91–4.54), 0.082 ^
CC	9 (34.6%)	63 (58.9%)	**0.37 (0.15–0.90), 0.029**	0.66 (0.29–1.50), 0.323
TT + CT	17 (65.4%)	44 (41.1%)	**2.70 (1.10–6.62), 0.029**	1.51 (0.67–3.43), 0.323
*IL-1B* allele				
Allele T	18/52 (34.6%)	51/214 (23.8%)	1.69 (0.88–3.25), 0.114	1.05 (0.59–1.87), 0.873
Allele C	34/52 (65.4%)	163/214 (76.2%)	0.59 (0.31–1.13), 0.114	0.95 (0.53–1.70), 0.873

Differences between groups were evaluated by OR and CI for categorical variables. Statistically significant differences were indicated in bold. Trends were denoted with ^.

**Table 5:** Genotype and allele frequencies of *IL-1B* rs16944 (-511) comparisons between patients with melanoma in lower limbs (n = 28), and patients with melanoma in other anatomical sites (n = 105), and healthy controls (n = 900).

	Lower Limbs Melanoma Patients (n = 28)	Other Sites Melanoma Patients (n = 105)	OR (CI), *p* Value, Lower Limbs vs. Other Sites Melanoma	OR (CI), *p* Value, Lower Limbs vs. Healthy Controls (n = 900)
*IL-1B* genotype				
TT	2 (7.1%)	6 (5.7%)	1.27 (0.24–6.66), 0.778	0.59 (0.14–2.52), 0.475
CT	16 (57.1%)	37 (35.2%)	**2.45 (1.05–5.73), 0.038**	1.70 (0.79–3.63), 0.173
CC	10 (35.7%)	62 (59.0%)	**0.39 (0.16–0.92), 0.031**	0.69 (0.32–1.52), 0.362
TT + CT	18 (64.3%)	43 (41.0%)	**2.60 (1.09–6.17), 0.031**	1.44 (0.66–3.15), 0.362
*IL-1B* allele				
Allele T	20/56 (35.7%)	49/210 (23.3%)	1.83 (0.97–3.44), 0.062 ^	1.10 (0.63–1.92), 0.736
Allele C	36/56 (64.3%)	161/210 (76.7%)	0.55 (0.29–1.03), 0.062 ^	0.91 (0.52–1.58), 0.736

Differences between groups were evaluated by OR and CI for categorical variables. Statistically significant differences were indicated in bold. Trends were denoted with ^.

**Table 6:** Genotype and allele frequencies of *IL-1B* rs16944 (-511) comparisons between patients with melanoma in lower extremities (n = 35), and remaining patients with melanoma in other anatomical sites (n = 98), and healthy controls (n = 900).

	Lower Extremities Melanoma Patients (n = 35)	Other Sites Melanoma Patients (n = 98)	OR (CI), *p* Value, Lower Extremities vs. Other Sites Melanoma	OR (CI), *p* Value, Lower Extremities vs. Healthy Controls (n = 900)
*IL-1B* genotype				
TT	2 (5.7%)	6 (6.1%)	0.93 (0.18–4.83), 0.930	0.46 (0.11–1.96), 0.296
CT	21 (60.0%)	32 (32.7%)	**3.09 (1.39–6.87), 0.005**	1.91 (0.96–3.80), 0.066 ^
CC	12 (34.3%)	60 (61.2%)	**0.33 (0.15–0.74), 0.007**	0.65 (0.32–1.33), 0.238
TT + CT	23 (6.6%)	38 (38.8%)	**3.03 (1.35–6.79), 0.007**	1.53 (0.75–3.12), 0.238
*IL-1B* allele				
Allele T	25/70 (35.7%)	44/196 (22.4%)	**1.92 (1.06–3.47), 0.031**	1.10 (0.67–1.81), 0.708
Allele C	45/70 (64.3%)	152/196 (77.6%)	**0.52 (0.29–0.94), 0.031**	0.91 (0.55–1.50), 0.708

Differences between groups were evaluated by OR and CI for categorical variables. Statistically significant differences were indicated in bold. Trends were denoted with ^.

**Table 7:** Comparison of demographic and clinical characteristics between groups of patients with melanoma localized in lower extremities (lower limb plus feet) (n = 35), and in the other anatomical sites (n = 98).

Characteristic	Lower Extremities Melanoma (n = 35)	Other Sites Melanoma (n = 98)	OR (CI), Lower Extremities vs. Other Sites	*p* Value, Lower Extremities vs. Other Sites
Age < 50 years at study enrolment, n (%)	7 (20.0%)	22 (22.4%)	0.86 (0.33–2.24)	0.763
Age at study enrolment, years, mean ± SD	62.5 ± 12.6	60.3 ± 12.9	-	0.398
Age < 50 years at melanoma diagnosis	12 (34.3%)	36 (36.7%)	0.90 (0.40–2.02)	0.796
Age at melanoma diagnosis, years, mean ± SD	55.5 ± 13.3	53.7 ± 13.9	-	0.510
Females, n (%)	21 (60.0%)	37 (37.8%)	**2.47 (1.12–5.45)**	**0.025**
Males, n (%)	14 (40.0%)	61 (62.2%)	**0.40 (0.18–0.89)**	**0.025**
All grandparents born in FVG	23 (65.7%)	73 (74.5%)	0.66 (0.29–1.51)	0.322
BMI kg/m^2^, mean ± SD	25.9 ± 3.61	25.8 ± 4.11	-	0.944
BMI ≥ 30 kg/m^2^, n (%)	4 (11.4%)	18 (18.4%)	0.57 (0.18–1.83)	0.347
Present smoker, n (%)	3 (8.6%)	9 (9.2%)	0.93 (0.24–3.64)	0.914
Ever smoker, n (%)	20 (57.1%)	45 (45.9%)	1.57 (0.72–3.42)	0.256
≥ 20 years smoking ever in all subjects, n (%)	12 (34.3%)	28 (28.6%)	1.30 (0.57–2.97)	0.527
≥ 20 cigarettes/day ever in all subjects, n (%)	7 (20.0%)	18 (18.4%)	1.11 (0.42–2.94)	0.832
Cups of espresso coffee per day ≥ 2, n (%)	29 (82.9%)	71 (72.4%)	1.84 (0.69–4.92)	0.226
Phototype 1 and 2, n (%)	22 (62.9%)	55 (56.1%)	1.32 (0.60–2.92)	0.489
Nevi ≥ 50, n (%)	18 (51.4%)	48 (49.0%)	1.10 (0.51–2.39)	0.804
Low/no tanner, n (%)	20 (57.1%)	54 (55.1%)	1.09 (0.50–2.37)	0.835
Burns ≥ 5 lifelong, n (%)	18 (51.4%)	53 (54.1%)	0.90 (0.42–1.95)	0.787
Burns ≥ 10 lifelong, n (%)	15 (42.9%)	34 (34.7%)	1.41 (0.64–3.10)	0.391
Indoor tanning ≥ 1 ever, n (%)	8 (22.9%)	22 (22.4%)	1.02 (0.41–2.57)	0.960
MeM, n (%)	20 (57.1%)	43 (43.9%)	1.71 (0.78–3.72)	0.179
Stage I, n (%)	12 (34.3%)	40 (40.8%)	0.76 (0.34–1.69)	0.497
Stage II, n (%)	3 (8.6%)	15 (15.3%)	0.52 (0.14–1.91)	0.324
Stage III, n (%)	12 (34.3%)	25 (25.5%)	1.52 (0.66–3.50)	0.322
Stage IV, n (%)	8 (22.9%)	18 (18.4%)	1.32 (0.51–3.37)	0.566
Superficial spreading, n (%)	13 (37.1%)	57 (58.2%)	**0.42 (0.19–0.94)**	**0.035**
Nodular, n (%)	12 (34.3%)	33 (33.7%)	1.03 (0.46–2.32)	0.948
Acral lentiginous, n (%)	4 (11.4%)	1 (1.0%)	**12.5 (1.35–116.2)**	**0.026**
Lentigo maligna, n (%)	1 (2.9%)	1 (1.0%)	2.85 (0.17–46.9)	0.463
Spitzoide, n (%)	2 (5.7%)	3 (3.1%)	1.92 (0.31–12.0)	0.486
Breslow thickness, mm, mean ± SD	2.25 ± 1.86	1.98 ± 1.86	-	0.454
Breslow thickness ≤ 0.75 mm, n (%)	10 (28.6%)	25 (25.5%)	1.17 (0.49–2.77)	0.724
Breslow thickness 0.76–1.00 mm, n (%)	1 (2.9%)	12 (12.2%)	0.21 (0.03–1.68)	0.142
Breslow thickness 1.01–2.00 mm, n (%)	10 (28.6%)	28 (28.6%)	1.00 (0.43–2.35)	1.000
Breslow thickness 2.01–4.00 mm, n (%)	9 (25.7%)	20 (20.4%)	1.35 (0.55–3.33)	0.515
Breslow thickness ≥ 4.01 mm, n (%)	5 (14.3%)	13 (13.3%)	1.09 (0.36–3.31)	0.880
Ulceration, n (%)	14 (40.0%)	37 (37.8%)	1.10 (0.50–2.42)	0.815
Mitosis ≥ 1 per mm^2^, n (%)	27 (77.1%)	56 (58.3%) ^b^	2.41 (0.99–5.85)	0.052 ^
Regression, n (%)	5 (14.3%)	15 (15.6%) ^b^	0.90 (0.30–2.69)	0.850
Brisk positive TILs ^a^, n (%)	9 (25.7%)	29 (30.2%) ^b^	0.80 (0.33–1.92)	0.616
Non-brisk TILs ^a^, n (%)	9 (25.7%)	38 (39.6%) ^b^	0.53 (0.22–1.25)	0.147
TILs ^a^ absence, n (%)	17 (68.0%)	28 (29.2%) ^b^	**2.29 (1.04–5.08)**	**0.041**
Microsatellitosis, n (%)	0 (-)	5 (5.2%) ^b^	0.23 (0.01–4.35)	0.330
Epithelioid variant, n (%)	10 (28.6%)	27 (27.8%) ^c^	1.04 (0.44–2.44)	0.934
Fusate variant, n (%)	1 (2.9%)	12 (12.4%) ^c^	0.21 (0.03–1.66)	0.139
Small cell variant, n (%)	0 (-)	2 (2.1%) ^c^	0.54 (0.03–11.5)	0.691
More than 1 melanoma, n (%)	6 (17.1%)	15 (15.3%)	1.16 (0.41–3.27)	0.781
Additional non-melanoma skin cancer, n (%)	6 (17.1%)	16 (16.3%)	1.07 (0.38–3.00)	0.893
Additional non-skin cancer, n (%)	10 (28.6%)	19 (19.4%)	1.66 (0.68–4.04)	0.261
Melanoma familiarity, n (%)	3 (8.6%)	15 (15.3%)	0.52 (0.14–1.91)	0.324
Dead ≤ 5 years from first diagnosis	4 (11.4%)	14 (14.3%)	0.77 (0.24–2.53)	0.672

Differences between groups were evaluated by OR and 95%CI for categorical variables and by Student’s *t*-test for independent samples for continuous variables. ^a^ TILs, tumor infiltrating lymphocytes. ^b^ Data were available for 96 patients. ^c^ Data were available for 97 patients. Significant differences were indicated in bold. Trends were denoted with ^.

**Table 8:** Genotype and allele frequencies of *IL-1B* rs16944 (-511) comparisons between upper-extremities (upper limbs + hands) melanoma patients (n = 10), and remaining patients with melanoma in other sites (n = 123), and healthy controls (n = 900).

	Upper Extremities Melanoma Patients (n = 10)	Other Sites Melanoma Patients (n = 123)	OR (CI), *p* Value, Upper Extremities vs. Other Sites	OR (CI), *p* Value, Upper Extremities vs. Healthy Controls (n = 900)
*IL-1B* genotype				
TT	0 (-)	8 (6.5%)	0.65 (0.03–12.0), 0.770	0.36 (0.02–6.24), 0.485
CT	2 (20.0)	51 (41.5%)	0.35 (0.07–1.73), 0.199	0.32 (0.07–1.51), 0.149
CC	8 (80.0)	64 (52.0%)	3.69 (0.75–18.1), 0.108	**5.00 (1.06–23.7), 0.042**
TT + CT	2 (20.0)	59 (48.0%)	0.27 (0.06–1.33), 0.108	**0.20 (0.04–0.95), 0.042**
*IL-1B* allele				
Allele T	2/20 (10.0%)	67/246 (27.2%)	0.30 (0.07–1.31), 0.110	**0.22 (0.051–0.95), 0.043**
Allele C	18/20 (90.0%)	179/246 (72.8%)	3.37 (0.76–14.9), 0.110	**4.55 (1.05–19.7), 0.043**

Differences between groups were evaluated by OR and CI for categorical variables. Statistically significant differences were indicated in bold.

## Data Availability

All data from this study are available upon reasonable request.
